# Editorial: Closing the loop: enhancing biotechnological routes for a more circular economy transition

**DOI:** 10.3389/fbioe.2025.1697004

**Published:** 2025-09-19

**Authors:** Cristina Campano, Virender Kumar, María José Fabra, Felice Quartinello

**Affiliations:** ^1^ Polymer Biotechnology Group, Biological Research Centre Margarita Salas, Spanish National Research Council (CIB-CSIC), Madrid, Spain; ^2^ Department of Chemistry and Bioscience, Aalborg University, Aalborg, Denmark; ^3^ Food Safety and Preservation Department, Institute of Agrochemistry and Food Technology (IATA), CSIC, Valencia, Spain; ^4^ ACIB GmbH, Tulln an der Donau, Austria; ^5^ Department of Agricultural Sciences, Institute of Environmental Biotechnology, BOKU University, Tulln an der Donau, Austria

**Keywords:** circular bioeconomy, biotechnology, waste valorization, enzymatic and microbial processes, sustainability

## The challenge ahead

The relentless growth of the global population, coupled with rising rates of material and energy consumption, has led to an unprecedented accumulation of solid waste worldwide (Chen et al., 2020). Managing these vast waste streams is not only an environmental necessity but also a pressing socio-economic challenge, as inadequate disposal contributes to pollution, greenhouse gas emissions, and the depletion of natural resources (Singh et al., 2014). Among the different waste categories, municipal solid waste represents one of the most complex and heterogeneous fractions (Voukkali et al., 2024; Zhang et al., 2024). It encompasses diverse materials such as food residues, textiles, fabrics, and synthetic polymers, whose mixed and contaminated nature makes separation, recycling, and valorization particularly difficult (Nanda and Berruti, 2021). This complexity severely limits the effectiveness of conventional waste management strategies and underscores the urgent need for innovative solutions capable of transforming such residues into valuable resources (Campuzano and González-Martínez, 2016; Xiao et al., 2024; Kumar et al., 2025).

Overcoming these challenges requires going beyond traditional recycling practices, which, while essential to a circular economy, are often insufficient to deal with the complexity and heterogeneity of modern waste streams (Awino and Apitz, 2024; Corvellec et al., 2022). To effectively reduce dependence on fossil-based resources, there is a pressing need to develop advanced strategies that combine improved energy and material efficiency with the creation of novel, high-quality bio-based products (Khanna et al., 2024). Such innovations are key to meeting the rising global demand for goods while ensuring environmental sustainability.

In this context, biotechnology represents a powerful driver of the transition towards a circular and resource-efficient economy (Schilling and Weiss, 2021; Vandy et al., 2025). By harnessing advances in bacterial microbiology and enzymatic catalysis, it is now possible to transform heterogeneous waste streams into a wide range of bio-based products, from biofuels to high-performance polymers (Aurand et al., 2024; Torres-León et al., 2021). Recent progress illustrates the breadth of this potential: novel microbial biocatalysts are being identified through OMIC analyses (Hassan et al., 2022; Parveen et al., 2022); innovative enzymatic routes are being developed for polymer recycling (Kumar et al., 2025; Lens-Pechakova, 2021; Zhu et al., 2022) and producing microorganisms or microbial consortia are being refined through metabolic engineering and adaptive laboratory evolution (Guzmán, 2023; Hernández-Herreros et al., 2024; Hirasawa and Maeda, 2022). These advances are complemented by the development and optimization of sustainable bioproduction processes, ultimately paving the way for novel microbial-based value chains that can substitute fossil-derived products (Mihalyi et al., 2024; Pardo et al., 2025). To ensure that these innovations deliver genuine environmental benefits, life cycle analysis (LCA) provides a critical benchmark, allowing bio-based products and processes to be systematically compared with their conventional fossil-based counterparts (Cucurachi et al., 2022). We are convinced that biotechnology will play a central role in enabling this transition, and it is with this perspective that we have curated this Research Topic, whose contributions are summarized in the following sections.

## Insights from the Research Topic

The articles included in this Research Topic collectively showcase the breadth of biotechnological approaches being developed to address pressing environmental and societal challenges. They span from enzymatic and microbial strategies for advanced recycling and waste valorization, to biotechnological innovations for renewable energy production, and the discovery of bioactive compounds with therapeutic potential. Together, these contributions highlight how biotechnology through its diverse applications in materials, energy, and health can open new pathways towards a more sustainable and circular bioeconomy. This Research Topic brings together four articles authored by 28 researchers, reflecting the collaborative effort driving progress in the field.

The contributions authored by Siracusa et al. focuses on the biochemical characterization of a novel polyester-hydrolyzing enzyme (Thb) derived from anaerobic *Thermoanaerobacterales*. This enzyme exhibits a pronounced specificity for aromatic polyesters compared to a well-known benchmark enzyme, revealing distinct potential for targeted polymer degradation. Through comparative analyses—including weight loss measurements and quantification of monomer release the study illuminates how structural differences in Thb underlie its enhanced activity on aromatic substrates. This work exemplifies how microbial catalysts can be leveraged to valorize plastic-rich waste streams and supports the development of more efficient, bio-based waste-to-resource processes.

A second contribution in the enzymatic recycling section of this Research Topic, authored by Egan et al. examines the use of commercial cellulase formulations to enable targeted enzymatic depolymerization of cotton within blended polyester/cotton textiles. By assessing a wide range of enzyme mixtures originally designed for biopolishing, stonewashing, or biomass degradation the study identifies enzyme activity profiles that optimize fiber separation in practical reactor systems. Importantly, the introduction of protein efficiency as a key metric links enzyme performance to economic viability, marking an important step toward scalable, enzyme-based recycling strategies. The results not only advance the scientific understanding of cellulase performance but also provide practical insights into how enzymatic tools can be adapted for more sustainable textile recycling practices.

More in the field of bioenergy, Chawla et al. explores microbial-based strategies to enhance renewable energy generation through the *in situ* biostimulation of coalbed methane (CBM) wells. Conducted at the Raniganj coal reservoir in India, this research demonstrates that nutrient-induced activation of endogenous microbial communities can quadruple methane production in certain wells. The study further examines how the microbial ecosystem shifts in response, offering insight into the bioconversion pathways at play.

Finally, the study developed by Abdelmalek et al. reports the purification and characterization of two novel protein-based α-amylase inhibitors from Saussurea costus, a medicinal plant traditionally associated with antidiabetic properties. The inhibitors demonstrated strong biochemical stability and inhibitory potency comparable to the standard drug acarbose, together with additional antimicrobial and anticancer activities. By uncovering the therapeutic potential of plant-derived molecules, the study broadens the scope of biotechnology toward health applications and highlights the value of bioprospecting in the circular bioeconomy.

## Final reflections

We are confident that this Research Topic will stimulate further research and collaboration at the interface of biotechnology, sustainability, waste management, and health. By bringing together diverse approaches from enzymatic recycling and microbial bioenergy to the discovery of bioactive compounds, this Research Topic reinforces the transformative role of biotechnology in closing the loop and demonstrates its potential to drive innovation toward a truly circular bioeconomy.
